# Spinal neuromodulation using ultra low frequency waveform inhibits sensory signaling to the thalamus and preferentially reduces aberrant firing of thalamic neurons in a model of neuropathic pain

**DOI:** 10.3389/fnins.2024.1512950

**Published:** 2025-01-17

**Authors:** Martyn G. Jones, Liam A. Matthews, Scott Lempka, Nishant Verma, James P. Harris, Stephen B. McMahon

**Affiliations:** ^1^Wolfson Sensory, Pain and Regeneration Centre, King’s College London, London, United Kingdom; ^2^Zenith Neurotech Ltd., King’s College London, London, United Kingdom; ^3^Department of Biomedical Engineering, University of Michigan, Ann Arbor, MI, United States; ^4^Biointerfaces Institute, University of Michigan, Ann Arbor, MI, United States; ^5^Department of Anesthesiology, University of Michigan, Ann Arbor, MI, United States; ^6^Presidio Medical Inc., San Mateo, CA, United States

**Keywords:** thalamus, ULF^™^ neuromodulation, spinal cord stimulation, inhibition, neuropathic, pain

## Abstract

**Introduction:**

Many forms of chronic pain remain refractory to existing pharmacotherapies and electrical neuromodulation. We have recently reported the clinical efficacy of a novel form of analgesic electrical neuromodulation that uses ultra low frequency (ULF^™^) biphasic current and studied its effects on sensory nerve fibers. Here, we show that in anesthetized rats, epidural ULF current reversibly inhibits activation of neurons in the thalamus receiving sensory spinothalamic input.

**Methods:**

In naïve, neuropathic and sham-operated rats, recordings of ongoing and evoked activity were made from thalamic neurons, targeting the ventral posterolateral (VPL) nucleus.

**Results:**

Responses to electrical stimulation of hind limb receptive fields were reduced in 25 of 32 (78%) neurons tested with lumbar epidural ULF neuromodulation. Cells preferentially responsive to low intensity stimulation were more likely to be found than cells responding to a range of stimulus intensities, or high intensity only; and low threshold responses were more likely to be inhibited by ULF than high threshold responses. On-going activity unrelated to hindlimb stimulation, observed in 17 of 39 neurons in naïve animals (44%), was reduced by lumbar epidural ULF current in only 3 of 14 (21%) neurons tested with ULF. By contrast, in rats with a well-characterized neuropathic injury, spinal nerve ligation (SNL), we found a much higher incidence of on-going activity in thalamic neurons: 53 of 55 neurons (96%) displayed firing unrelated to hindlimb stimulation. In this group, ULF current reduced thalamic neurone discharge rate in 19 of 29 (66%) neurons tested. In sham-operated animals, the incidence of such activity in thalamic neurons and the effect of ULF current were not significantly different from the naïve group.

**Discussion:**

We conclude firstly that ULF current can acutely and reversibly interrupt signaling between sensory afferent fibers and relay neurons of the thalamus. Second, ongoing activity of thalamic neurons increases dramatically in the early stages following neuropathic injury. Third, this novel form of neuromodulation preferentially attenuates pathological thalamic activity in this neuropathic model compared to normal activity in naïve and sham-operated animals. This study, therefore, demonstrates that epidural ULF current can reduce nerve injury-related abnormal activity reaching the brain. These findings help advance understanding of possible mechanisms for the analgesic effects of ULF neuromodulation.

## Introduction

The challenge of treating chronic pain remains considerable. Some estimates place the incidence of chronic pain at 40% or more (Dahlhamer et al., 2018; Fayaz et al., 2016; Cohen et al., 2021) and the most recent Global Burden of Disease data indicate that musculoskeletal pathologies, including lower back pain and osteoarthritis, are particularly prevalent worldwide (GBD 2019 Diseases and Injuries Collaborators, 2020). Accelerated by the poor outcomes, side-effect profiles, and addiction problems (e.g., Scholl et al., 2018) associated with pharmacological therapy, coupled with the lack of progress in delivering novel analgesic drugs, the use of neuromodulation has expanded as an alternative strategy for pain relief (Knotkova et al., 2021).

Since its introduction more than 50 years ago (Shealy et al., 1967), conventional spinal cord stimulation (SCS) has been used to treat chronic pain. Stimulating current pulses delivered via epidural electrodes at a moderate tonic frequency of 40-70 Hz are intended to activate large-diameter, myelinated Aβ fibers that typically carry information for non-painful stimuli such as touch and pressure. This activation eventually leads to inhibition of incoming signals from small-diameter, nociceptive, weakly myelinated Aδ or unmyelinated C fibers, as predicted by the Gate Control theory of pain (Melzack and Wall, 1965). Thus, by activating Aβ fibers, conventional SCS generates concomitant paraesthesia over the appropriate locus of pain, with the aim of reducing nociceptive signaling in the dorsal horn of the spinal cord via activation of low-threshold, non-nociceptive elements (Taylor et al., 2014).

Novel forms of SCS, including burst and high-frequency paradigms, have been developed in an attempt to generate paraesthesia-free reduction of pain signaling and improve outcomes (Kapural et al., 2015; Deer et al., 2018; Lempka and Patil, 2018). In contrast to these stimulation strategies, which predominantly utilize high frequency and/or short pulse width trains, we have recently described a novel form of neuromodulation that employs an ultra-long pulse, ultra low frequency (ULF) biphasic current waveform (Jones et al., 2021). Each phase of the ULF waveform uses a slowly ramped rise, avoiding neural excitation, to a plateau phase, several seconds in duration and directly inhibits the passage of action potentials along axons. In our laboratory experiments in anesthetized rats, we showed that this waveform produces a rapidly-developing, fully reversible conduction block in sensory nerve fibers excited by peripheral stimulation. Our modeling work described how this inhibition is achieved immediately by inactivation of sodium channels during the plateau phases of the waveform. The slow ramped rise to the plateau phase avoids activation of axons. We also identified a delayed conduction block, developing over several minutes, that arises from profound changes in intracellular and extracellular potassium ion concentrations, leading to a depolarising shift and prolonged action potential block. In clinical studies, the ULF waveform is highly effective in reducing pain scores in patients suffering chronic back or leg pain (Jones et al., 2021).

With conventional metal electrodes, such long current biphasic pulses, approximating to short periods of direct current, would be damaging to neural tissue. However, the use of electrodes composed of material with a very high charge capacity circumvents any such effects. Such material allows the ultra long biphasic pulses to follow the primary rules of traditional biphasic neuromodulation pulses to avoid tissue or electrode damage (i.e., avoid the voltage range that causes water electrolysis and electrode potentials that would cause metal dissolution). The waveform has undergone extensive bench and animal testing to examine these risks and establish stability (Harris et al., 2022; Verma et al., 2025). ULF neuromodulation, therefore, differs fundamentally from other approaches that employ very short pulses designed to stimulate fibers within the spinal dorsal horn. To further characterize the effects of ULF neuromodulation, it is critical to investigate its impact at higher levels of the neuraxis.

There is considerable evidence of abnormal spontaneous activity in sensory fibers in patients with chronic pain. This activity appears to be important not only in the initiation of central sensitisation, but also in the maintenance of the sensitized state and in the generation of spontaneous pain—one of the most troublesome features associated with many types of chronic pain (Finnerup et al., 2021; Roza and Bernal, 2022). Accordingly, there is also evidence that chronic pain can be transiently relieved by suppression of activity in peripheral sensory fibers (Kuner and Flor, 2016).

**Figure 1:**
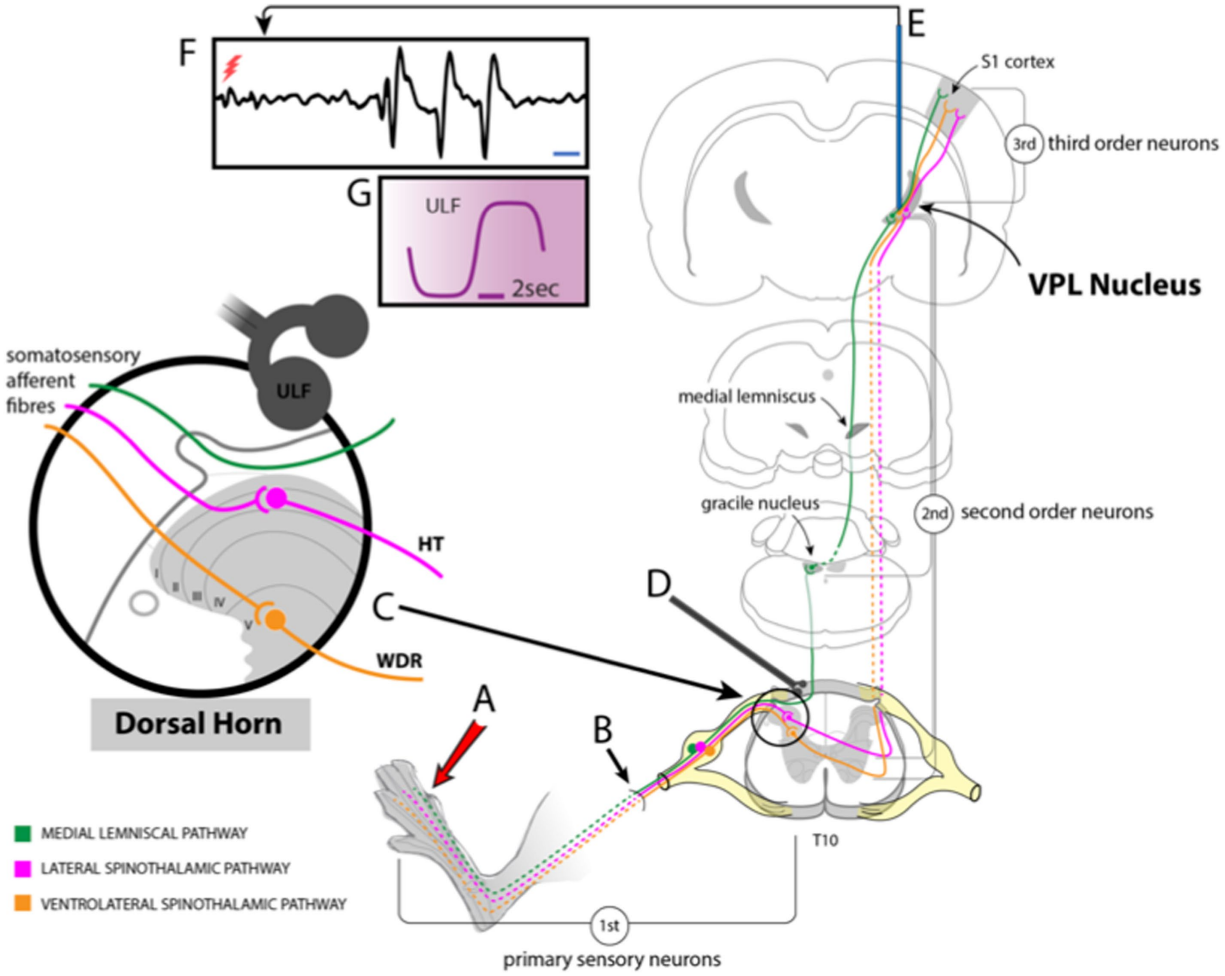
Schematic drawing showing experimental setup. **(A)** Stimuli applied to the hind paw activate sensory fibers **(B)** projecting to the dorsal horn of the spinal cord. Some of these fibers ascend in the ipsilateral dorsal column to contact 2nd order neurones in the gracile nucleus of the brainstem. Axons of those neurons cross and ascend in the medial lemniscal pathway to the VPL nucleus of the thalamus. Other fibers synapse with dorsal horn neurones **(C)** that project via the lateral or ventrolateral spinothalamic tracts to VPL. **(D)** ULF current is applied via a pair of epidural electrodes positioned just lateral to the spinal midline. **(E)** Tungsten microelectrodes were stereotactically inserted into the VPL to record thalamic neuron responses **(F)** to stimulation of peripheral receptive fields or spontaneous activity (red flash mark in F indicates stimulus timing). **(G)** Detail of ULF current waveform.

Under normal conditions, somatic nociceptive signals, processed at the spinal level, are relayed to the brain predominantly via neurons projecting through lateral and anterolateral tracts (ventral and ventrolateral in animals). Many axons ascending in these tracts terminate in the thalamus, where further signal processing occurs and determines if the information is relayed on to the somatosensory cortex. Thus, the thalamus represents a key relay station for pain signals ascending from the spinal level to higher brain centers (Figure 1). Under conditions of chronic pain arising from disease or injury, the total amount of sensory input arriving at the thalamus is likely to be elevated, amplifying thalamic neurone activity (Guilbaud et al., 1990; Patel and Dickenson, 2016) and thereby increasing thalamic signaling to other brain areas, including the sensory cortex.

In this study, therefore, our aims were twofold. Given the recognized importance of thalamic processing in the ascending transmission of sensory signals, we first wished to determine whether epidural ULF neuromodulation could acutely impact thalamic neurone responses to signals generated by stimulation of peripheral sensory receptive fields under normal conditions. Second, we wanted to examine the effect of epidural ULF current on previously reported pathological changes in thalamic activity (Guilbaud et al., 1990; Patel and Dickenson, 2016) in a model of neuropathic pain.

## Methods

### Animals

Male Sprague–Dawley rats (220-350g; Harlan, UK) were housed in the Biological Services Unit at King’s College London, in groups of three on a 12-h light–dark cycle with *ad libitum* access to food and water. Experimental procedures, following ARRIVE guidelines (Rice et al., 2008), were approved by the local ethical committee at the Wolfson CARD, King’s College London, and were performed according to UK Home Office regulations.

### Neuropathic pain model

Rats were anesthetised using a mixture of ketamine (90 mg/kg) and medetomidine (0.9 mg/kg) injected intraperitoneally (ip). The left L5 spinal nerve was carefully exposed, tightly ligated with 6/0 silk suture, and cut distally to the ligation site, approximately 5 mm from its entry point into the L5 vertebra. In sham-operated animals, the nerve was exposed but not ligated or cut. The wound was closed in layers using Vicryl suture and stainless-steel wound clips and the animals were allowed to recover naturally from the anesthetic in a warm recovery pod. Animals received post-surgical analgesia (meloxicam, 1 mg/kg ip) and a subcutaneous injection of glucose in saline to aid fluid replacement, before being returned to their home cages when fully conscious.

### Preparation of animals for electrophysiology

Rats were initially anesthetised with either urethane (1.5 g/kg ip) or sodium pentobarbitone (50–60 mg/kg ip). Polythene cannulae were secured in trachea, jugular vein, and carotid artery for the purpose of clear airway maintenance, administration of supplemental fluid or anesthetic, and monitoring of systemic blood pressure, respectively. The electrocardiogram was recorded via needle electrodes inserted into the skin of the forelimbs. The core temperature of the animals was maintained at 37 ± 1°C by means of an homœostatically controlled heating blanket (Harvard Apparatus, UK). Supplemental anesthetic was administered to pentobarbitone-anesthetized animals at a rate of approximately one fourth of the initial dose per hour, delivered by infusion pump. Dose rate was adjusted to maintain an adequate depth of anesthesia based on observations of blood pressure, heart rate, ventilation rate, withdrawal reflex to toe pinch and corneal blink reflex.

A laminectomy was performed to expose the 4th-6th lumbar spinal segments and the exposed area was covered with a layer of warm, fluid agar solution (4% in 0.9% saline). A rectangular block was cut out of the solidified agar and the resulting well was filled with warmed mineral oil to provide electrical insulation and prevent tissue drying. The head was stabilized in a stereotaxic frame and a mid-line incision made in the scalp to allow visualization of the parietal bones and Bregma. A craniotomy was performed and the dura overlying the brain was incised and reflected to permit the insertion of recording microelectrodes. The exposed cortical surface was covered with a film of mineral oil.

### Electrophysiology

Glass-coated tungsten microelectrodes with impedance 1–4 MΩ (Microelectrodes Ltd., Cambridge, UK) were inserted on vertical tracks to target the ventral posterolateral (VPL) nucleus of the thalamus, using coordinates from the rat brain atlas of Paxinos and Watson (2004). This area of the thalamus receives sensory input from the hind limb. Electrodes were inserted initially to a depth of 4.5 mm below the pial surface and then advanced in small increments of 10–20 μm while brushing, stroking, or tapping the receptive territory of the contralateral hind limb, particularly the plantar aspect of the paw. Stronger mechanical search stimuli, such as probing with firm pressure or brief pinching with broad-tipped metal forceps, were used very sparingly to minimize sensitisation.

When a cell was located that had an acceptable signal-to-noise ratio (generally minimum 2:1) and responded reliably to mechanical stimulation, a pair of pin electrodes was applied to the receptive field and percutaneous electrical stimulus pulses were applied to determine the activation threshold and latency of the thalamic unit’s response. Units were characterized both by their responses to electrical stimulation and to a series of simple tests for mechanosensitivity. Cells that responded only or preferentially to low intensity stimulation (i.e., brushing or stroking with a soft paint brush or cotton bud and/or electrical stimulation with a pulse width of 0.5–2 msec and amplitude ≤1 mA) were classed as low threshold (LT) cells. Those responding preferentially to high intensity stimulation (i.e., probing with a wooden probe of tip diameter 2 mm, brief pinching with metal forceps, and/or electrical stimulation with a pulse width of 2-3 msec width and amplitude of 1–5 mA) were classed as high threshold (HT) cells. Cells that responded with graded increases in firing rate to stimuli of both low and high intensity were regarded as wide dynamic range (WDR)-type.

The setup for stimulation and recording is schematically illustrated in Figure 1. Recordings in neuropathic or sham-operated rats were made 4–8 days post-surgery. Neural signals were amplified (x2k–x5k), conditioned (bandpass 300 Hz–3 kHz, 50 Hz removal) and digitized (20 kHz sampling rate) for computer display and storage using Neurolog equipment (Digitimer, Welwyn Garden City, UK), a Humbug 50 Hz signal conditioner (Quest Scientific, Vancouver, Canada) and a Power1401 interface with Spike 2 software (Cambridge Electronic Design, Cambridge, UK).

### ULF current

ULF current was applied using a pair of custom-made monopolar electrodes. Silver ball electrodes were formed by heating 0.5 mm diameter silver wire to melting point. The diameter of the silver ball thus formed was approximately 0.75 mm. These ball tips were chloride-coated and dipped into an electro-conductive agar solution, made with 0.9% saline, to form a solid tip droplet of approximately 1.0–1.5 mm in diameter. The electrodes were separated by a rostrocaudal gap of approximately 2 mm and were placed in contact with the dura mater overlying the spinal cord, initially at the level of the L4 and L5 dorsal root entry zones. The electrodes were positioned lateral to the cord midline on the side ipsilateral to the stimulated hind limb and contralateral to the thalamic recording site. We thus aimed to focus the ULF current more on the rootlets conveying primary afferent fibers to the root entry zone and over the dorsal column, through which many primary afferents ascend. Current was applied as a biphasic, ULF waveform, as illustrated in Figure 1. The ULF electrodes and cabling were shielded such that artifacts in the thalamic neurone recording signals resulting from ULF current applied at spinal level were insignificant.

When examining the effects of ULF current on evoked thalamic neurone firing, baseline responses to repeated hind paw electrical stimulation were observed before current was applied. Preliminary investigation revealed that these effects could be observed at amplitudes as low as 100 μA. Therefore, 100 μA current was usually chosen as a starting level and thereafter increased in 100 μA steps. Alternatively, when ULF effects on ongoing activity were tested, a baseline period of activity with no stimulation was recorded before ULF current was commenced. During application of the ULF currents, the ULF electrodes cycled from anode to cathode over a fixed period of 12 s. The ULF waveform, consisting of a non-linear ramp up, a plateau period and non-linear ramp down, has been described in detail previously (Jones et al., 2021). The waveform was generated by a custom-built apparatus (Presidio Medical, Inc., San Mateo, CA, USA). The device was adapted to drive an isolated biphasic stimulator (DS4, Digitimer, UK) that provided the current.

### Data analysis

To determine if ULF affected the stimulus-evoked responses, the mean number of spikes generated in response to a stimulus was calculated over a period of baseline and compared to that observed during plateau phases of ULF current. A reduction of response magnitude of ≥25% from baseline was considered as indicative of a genuine change, due to the ULF current. To assess the effect of ULF current on ongoing discharge rate, spike counts during a baseline period were compared with an equal period of ULF current. As above, a reduction of ≥25% from baseline was considered likely to be significant.

For changes in the range of 25–50%, evoked responses or counts of ongoing activity during baseline and during ULF current were compared using Student’s t-test. To compare ULF effects at the anodal vs. cathodal plateau phases the Wilcoxon Matched Pairs Signed Rank test was used. Elsewhere, as detailed in the Results, t-tests, Kruskal-Wallis analysis of variance, or Fisher’s Exact test were applied to determine significant differences. *p* < 0.05 was considered to represent significance. All statistical tests were performed using GraphPad Prism software (v. 9.5.1 or later).

## Results

Recordings were made from a total of 131 thalamic neurons in 33 rats. Recording sites of some of these neurons are shown in Supplementary Figure S1. Most cells recorded in the VPL fell into the LT classification, responding preferentially to low intensity electrical or mechanical stimulation. Fewer HT cells, responding only to high intensity stimulation, were encountered; and an even smaller cohort of WDR-type cells was found.

### Effect of ULF current on stimulus evoked responses in naïve animals

**Figure 2:**
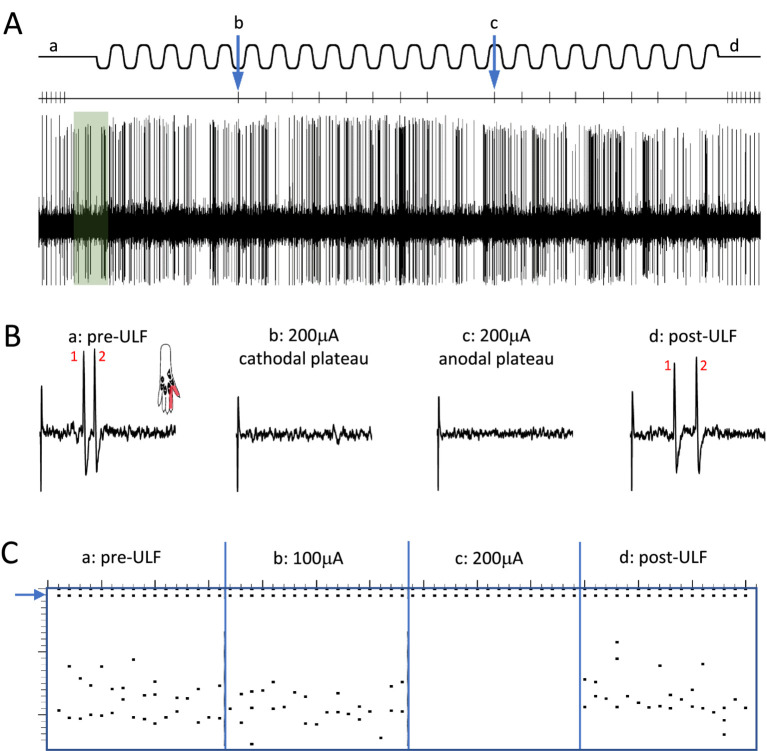
Epidural ULF applied at the level of the L4-L5 spinal segments reversibly inhibits foot-shock evoked low-threshold activity of a thalamic neurone. **(A)** Continuous recording from a thalamic unit responsive only to low-intensity electrical and mechanical (not shown) stimulation, with some ongoing spontaneous activity. Above the neurogram, the timing of electrical stimuli is indicated, with the ULF current waveform shown at the top of the panel. Prior to ULF current **(Aa)**, stimuli were applied at a constant rate of 1 Hz, usually evoking a pair of spikes. After 1 min of ULF current at 200 μA, stimuli were applied manually during the cathodal **(Ab)** and anodal **(Ac)** plateau phase of the ULF current. After switching off ULF, the constant 1 Hz stimulation was resumed **(Ad)**. Ongoing spontaneous activity of the neurone seen at baseline (green shaded box) after the foot stimulus is discontinued, and throughout the recording, was unaffected by the ULF current. **(B)** Example sweeps showing details of the foot stimulus-evoked spikes at **(Aa–Ad)**. At baseline, before ULF current **(Ba)**, the neurone fires a pair of spikes in response to an electrical foot shock delivered to its receptive field (shaded area in inset). A foot stimulus applied during the cathodal **(Bb)** and anodal **(Bc)** plateau phases of the ULF current (200 μA), respectively, evokes no response of the thalamic neurone. When the ULF current is discontinued, the neurone again fires a pair of spikes in response to the resumed foot stimulus **(Bd)**. **(C)** Raster plot compiled from a previous ULF application in the same cell where the current was applied at 100 μA and increased to 200 μA. Dots at the top (blue arrow) indicate stimulus artifacts; dots below indicate the occurrence of spikes, usually in pairs, with some jitter in the interspike interval. Plot shows absence of effect of ULF current at 100 μA, instant and complete inhibition of response at 200 μA, and immediate recovery when current is switched off (vertical scale at left, 0-25 msec post-stimulus).

ULF current inhibition of thalamic neurone responses to stimulation of receptive fields was seen across all cell types. Effects of ULF current on electrically evoked responses were tested in 32 of 39 cells from naïve animals. ULF current was tested against LT electrically evoked responses (spike latency 10–50 msec) in 22 LT cells responding only to low-intensity stimulation, and in 5 WDR cells (total n = 27 LT tests). Similarly, the effect of ULF was tested against HT electrically evoked responses (spike latency >100 msec) in 4 of the 5 WDR cells and in 5 HT cells that responded only to high-intensity stimulation (total *n* = 9 HT tests). ULF current was apparently more effective in attenuating responses evoked by low-intensity stimulation. However, the small sample size mandates caution in interpretation of this difference. Table 1 summarizes the effects of ULF current on LT and HT evoked responses to electrical stimuli. Examples of this effect are shown in Figure 2 and Supplementary Figure S2.

**Table 1:** Summary of ULF current effects on electrically-evoked responses in Naïve rats.

Response type	Sum	Unaffected	Inhibited	% Inhibited
LT	22	2	20	91
HT	9	5	4	44

LT includes cells that responded fully to low intensity stimuli; HT includes high-threshold responses of WDR-type cells together with cells responding to high intensity stimuli only. Fisher’s Exact test, *p* = 0.012.

**Figure 3:**
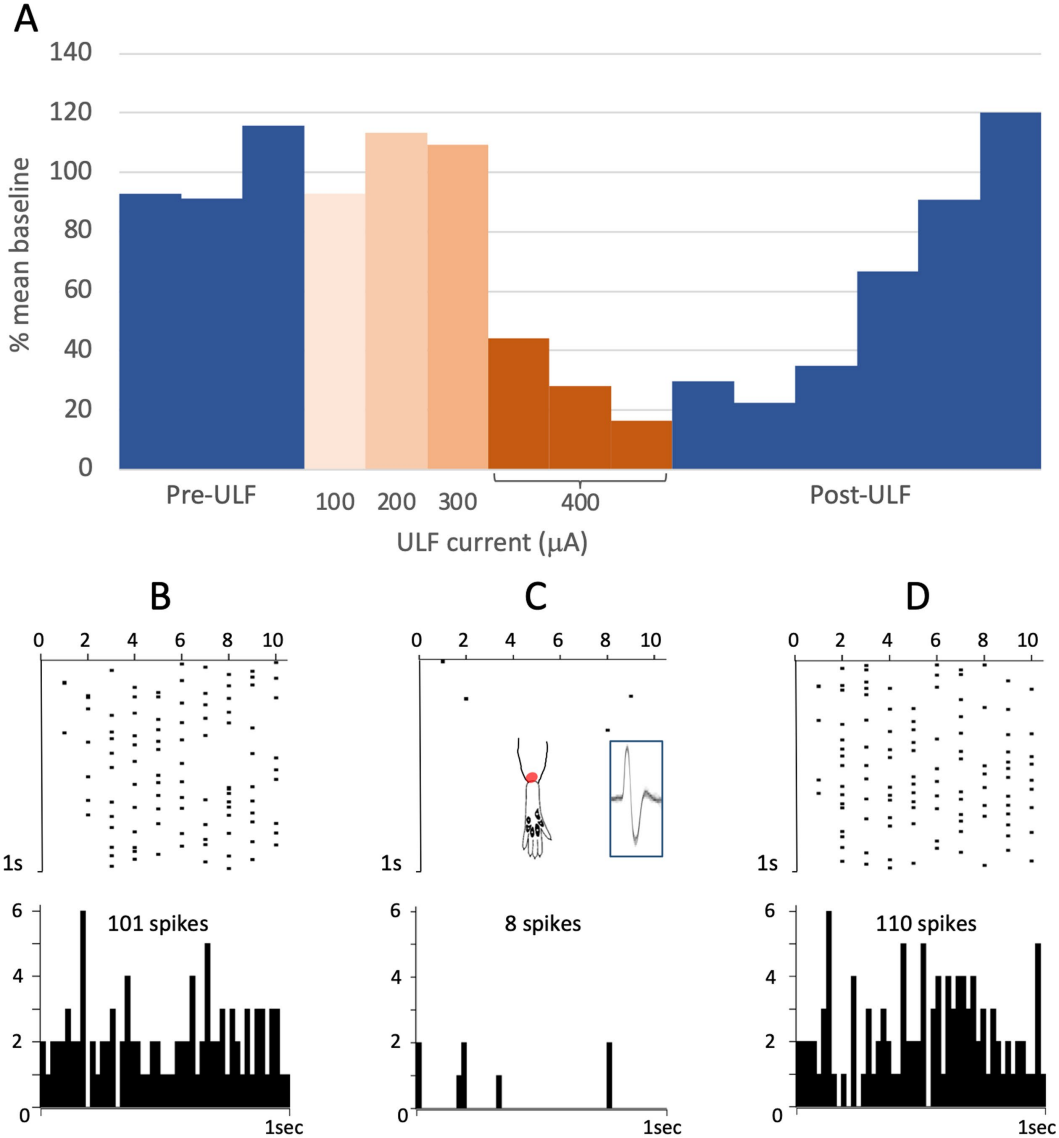
ULF current reversibly attenuates cumulative response of an HT thalamic neurone to a sequence of electrical stimuli. The neurone was unresponsive at low intensity stimulation. High intensity pulses (2.0 msec, 3.4 mA, 2 pulses at 200 Hz) applied percutaneously via a pair of pin electrodes placed in the receptive field (lateral ankle) at a rate of 1 Hz evoked a cumulative response with many spikes at longer latency (100–1000 msec post-stimulus) and an after-discharge of variable duration. **(A)** Bars in chart show the cumulative responses of the neurone (spike count at 0–1000 msec post-stimulus plus 20 s after-discharge) to a sequence of 10 electrical stimuli. After stable baseline responses were recorded, ULF current was applied epidurally at spinal level L5-L6. No reduction in the cumulative response was seen at currents of 100–300 μA. After 12 min at 400 μA, the spike count was progressively reduced to 8% of the mean baseline. On discontinuation of the ULF current, response magnitude recovered fully over a period of 30 min. **(B–D)** Raster plots showing example responses at baseline **(B)**, at 400 μA ULF **(C)**, and at 25 min post-ULF **(D)**. Insets on **(C)** show receptive field of neurone near ankle and overlay of 20 spikes recorded during a baseline stimulus sequence.

When the effects of ULF current on LT responses in LT cells were compared with effects on HT responses in WDR and HT cells combined, the evoked responses of LT neurons were more likely to be inhibited than HT responses of either WDR or HT neurons (Fisher’s Exact test, *p* = 0.023; Table 1). Again, we interpret this difference with caution due to the small sample size. Figure 3 and Supplementary Figure S3 illustrate, respectively, examples of the reduction of evoked responses of an HT and a WDR type neurone by ULF current. In some tests, increasing inhibition of responses was seen with increasing magnitude or duration of ULF current, and full recovery of responses to baseline could take several minutes (e.g., Supplementary Figure S2).

In some instances, we noted different levels of inhibition when the epidural ULF current electrodes were placed at different rostrocaudal locations, though this effect was not particularly strong in our model (Supplementary Figure S4). Overall, in cells that were inhibited by ULF current, we found no significant difference between the observed changes in responses during the cathodal versus anodal plateau phases of the ULF waveform (*p* > 0.05, Wilcoxon Matched Pairs Signed Rank test; Supplementary Figure S5).

### Incidence of ongoing firing in neuropathic rats vs. sham controls and naïve rats

**Figure 4:**
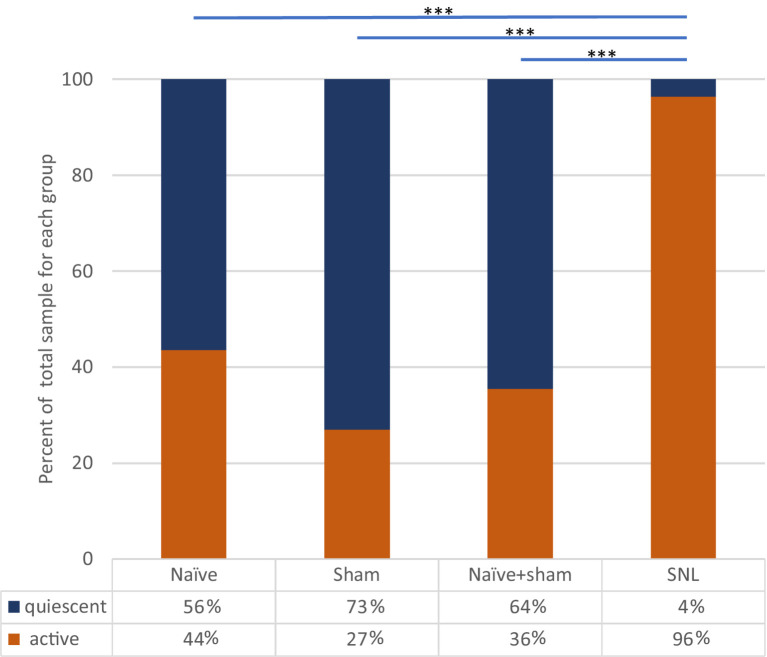
Incidence of spontaneously active thalamic neurons in each experimental group as a percentage of the whole group sample. Bars and significance levels are shown to indicate group comparisons with Fisher’s Exact test, using cell numbers from Table 2. ***p < 0.0001.

In SNL rats, the proportion of thalamic neurons recorded with ongoing activity unrelated to stimulation was significantly higher than that seen in either the Sham group (*p* < 0.0001, Fisher’s Exact test) or in naïve animals (*p* < 0.0001). The cell numbers of all groups are presented in Table 2 and Figure 4. To ensure that this result was not inadvertently biased by increased sampling in one group compared to another, we compared the number of electrode tracks made per animal. In the Sham group, the number of tracks made per experiment was 5.3 ± 2.0 (mean ± SEM) which was significantly more than in the naïve group (3.1 ± 0.7, *p* = 0.02), though not greater than in the SNL group (3.8 ± 1.3). However, when active units per electrode track were compared, the number for SNL animals was significantly higher at 2.6 ± 0.9 active cells/track, versus 0.4 ± 0.1 in Naïve animals (*p* = 0.0011) and 0.4 ± 0.2 in Sham animals (*p* = 0.019; all comparisons used Kruskal Wallis analysis of variance with Dunn’s multiple comparison test), suggesting that active cells were more readily found in the SNL group (Supplementary Figure S6). There was no significant difference in the firing rates of active cells in any of the treatment groups (Naïve, 3.0 ± 0.9 spikes/s, range 0.1–13.0, median 1.5; Sham, 2.4 ± 2.0, range 0.1–5.8, median 2.0; SNL, 3.1 ± 0.7, range 0.3–22.0, median 1.2).

**Table 2:** Numbers of quiescent and spontaneously active thalamic neurons recorded in the three experimental groups.

Treatment group	Total cells	Quiescent (% of total)	Spontaneous (% of total)
Naïve	39	22 (56%)	17 (44%)
Sham	37	27 (73%)	10 (27%)
Naïve + Sham	76	49 (64%)	27 (36%)
SNL	55	2 (4%)	53 (96%)

The proportion of spontaneously active cells was significantly higher in the SNL rats compared to either the naïve or sham groups, or the combined numbers from these two non-neuropathic groups (*p* < 0.0001, Fisher’s Exact test, all comparisons). There was no significant difference between the Naïve and Sham groups (*p* = 0.156).

### Effect of ULF on ongoing firing in neuropathic rats vs. sham controls and naïve rats

**Figure 5:**
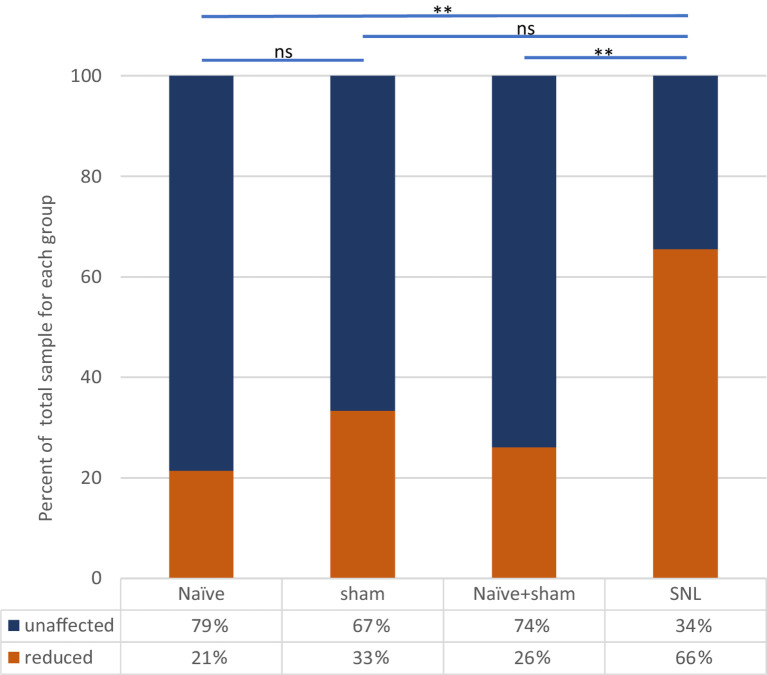
Effect of ULF current on spontaneous firing of thalamic neurons in each experimental group. Bars and significance levels are shown to indicate group comparisons with Fisher’s Exact test, using cell numbers from Table 3. **p < 0.01; ns, not significant (*p* = 0.128).

**Figure 6:**
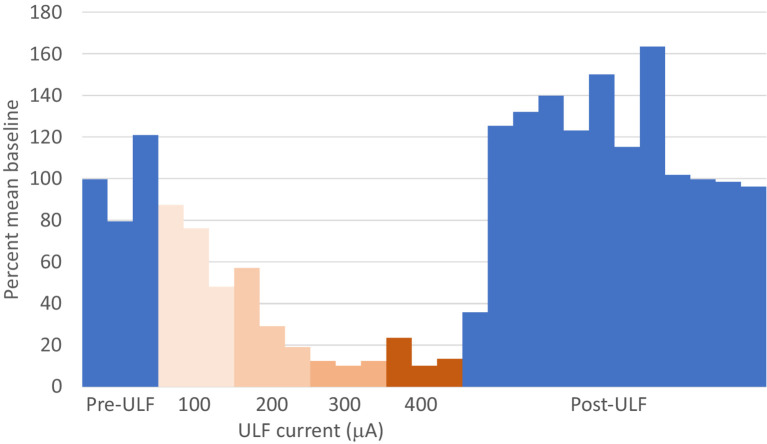
ULF current reversibly inhibits spontaneous firing in a thalamic neurone following L5 spinal nerve injury. Bars represent 1 min epochs. Numbers below chart indicate ULF current in μA. Firing rate recovered rapidly, and this cell appeared to exhibit a rebound increase in firing rate after ULF current was discontinued. Rate returned to pre-ULF baseline 9 min after ULF current was switched off.

Clear differences were observed between the different groups of animals in the effect of spinal epidural ULF current on ongoing firing of thalamic neurons (Figure 5 and Table 3). ULF current was more likely to reduce the rate of thalamic neurone firing in SNL rats compared to the non-injured groups—either Naïve alone (*p* = 0.005, Fisher’s Exact test) or Naïve+Sham numbers combined (*p* = 0.003). There was a numerical difference between the SNL and the Sham group alone, though this difference did not reach significance (*p* = 0.128), perhaps because of the smaller size of the Sham cohort. An example of the effect of increasing ULF amplitudes on the firing of a thalamic neurone 6 days after L5 SNL is shown in Figure 6.

**Table 3:** Numbers of spontaneously active thalamic neurons showing either reduction in rate by ULF current or unaffected.

Treatment group	Cells tested with ULF	Unaffected by ULF (% unaffected)	Reduced by ULF (% reduced)
Naïve	14	11 (79%)	3 (21%)
Sham	9	6 (67%)	3 (33%)
Naïve + Sham	23	17 (74%)	6 (26%)
SNL	29	10 (34%)	19 (66%)

The proportion of active cells slowed by ULF current was significantly higher in the SNL rats compared to either the naïve (*p* = 0.01) or the numbers from the two non-neuropathic groups combined (naïve + sham, *p* = 0.006) but not compared to the sham group alone (*p* = 0.128). There was no difference between the Naïve and Sham groups (*p* = 0.643, Fisher’s Exact test, all comparisons).

In the cohort of 76 cells recorded in the combined Naïve+Sham group, we found that 36% (27/76) of the units showed some level of ongoing firing (Table 2) and only a small proportion of cells in this group, where ULF current was tested (6/23), showed a firing rate reduction (Table 3). By contrast, in SNL animals, 66% (19/29) of cells responded during ULF current with a firing rate reduction. Thus, only 34% of cells with ongoing activity in the SNL group did *not* respond to ULF current (Table 3), suggesting that those non-responsive cells are proportionally similar in number to that encountered in non-injured animals. When the proportion of active cells in uninjured animals was compared with the proportion of ULF-non-responsive active cells in SNL animals, there was no significant difference (*p* < 0.99, Fisher’s Exact test). ULF current, therefore, appears to have a normalizing effect on the heightened ongoing thalamic neuron activity in the SNL group.

## Discussion

The experiments described here demonstrate that ULF current, applied epidurally over the spinal cord, reduces evoked responses of thalamic neurons to stimulation of peripheral receptive fields in the non-pathologic state and preferentially reduces ongoing, aberrant firing in the pathologic state. Whilst it remains uncertain upon which spinal neural elements ULF current is exerting its primary effect, these results cement our previous findings that ULF neuromodulation produces a dose-dependent inhibition of the nervous system and continues to support the utility of ULF current for the reduction of chronic pain signals.

Our previous study described for the first time the inhibitory effect of the novel ULF waveform on conduction of action potentials along primary sensory afferent fibers, such that both evoked action potentials in normal fibers and ectopic spontaneous impulses in axotomised fibers could be prevented from reaching the spinal cord (Jones et al., 2021). While others have suggested that long pulse widths may have differential effects on different ion channels (Yang et al., 2018) leading to a potential mechanism to affect normal vs. pathological fibers, we have not examined this aspect (normal vs. pathological ion channel expression and ULF’s effects thereon) in this work. Our previous computational studies demonstrated that the ULF waveform results in sodium channel inactivation and extracellular potassium accumulation in fibers close to the ULF electric field, effectively preventing the passage of action potentials. We showed that larger diameter, myelinated, faster-conducting fibers are more susceptible to inhibition by ULF current. To further investigate this finding, we wanted to understand the supraspinal effects that may follow from ULF inhibition at the spinal level.

As the thalamus is an important waystation on the route of sensory signals to the cortex, we investigated how ULF current applied at spinal level may alter supraspinal signaling. In our findings, we achieve proof of principle in showing that ULF current, applied epidurally at lumbar cord level in laboratory animals, impacts transmission of signals from a variety of peripheral sensory receptor types and fields to brain centers. We show this to be the case in both normal healthy animals and in a model of nerve injury, where sensory input to the thalamus is pathologically enhanced. Why is this important?

Many reports have described changes in thalamic neurone sensitivity and activity in models of chronic pain, including nerve injury (Guilbaud et al., 1990; Vos et al., 2000; Syré et al., 2014; Patel and Dickenson, 2016), diabetic neuropathy (Fischer et al., 2009), polyarthritis (Gautron and Guilbaud, 1982), inflammation (Guilbaud et al., 1986) and spinal cord injury (Miki et al., 2000; Hains et al., 2005, 2006). In such cases, including the SNL model used in this study, where damaged sensory afferent fibers have developed spontaneous discharge, at least some of this peripherally generated afferent signal is likely to reach the brain via ascending spinal pathways. Activity originating in pathological nociceptive fibers may be relayed to the thalamus via second-order nociceptive-specific or WDR-type neurons projecting in the spinothalamic tracts. Alternatively, the dorsal column-lemniscal pathway may provide a route for ectopic action potentials arising in normally non-nociceptive sensory fibers that become active in disease or following injury (Miki et al., 1998b; Miki et al., 2000).

Let us first consider the prevalence of different response types observed in our experiments and the effect of ULF current on evoked thalamic activity under normal, non-pathological conditions. The relative proportions of LT neurons to HT neurons reported in different studies varies considerably. Some studies have reported somewhat higher incidence of HT neurons than we encountered (Guilbaud et al., 1980; Casey and Morrow, 1983; Chung et al., 1986). There are multiple factors that may account for this difference, including presence of anesthetic (some groups have recorded only in awake animals), type of anesthetic, species investigated, and the search method used to locate neurons. Our view is that the search method is likely to be of primary importance with respect to our findings. We took care to apply high-intensity stimuli very sparingly to minimize the likelihood of sensitizing the skin or deeper tissues by repeatedly pinching the toes, for example. Thus, whilst most cells with LT responses were also tested to see if they responded to more intense stimuli (i.e., WDR-type), we seldom employed high-intensity stimuli as a primary search stimulus. This may have biased our sample to include more LT neurons. With this approach, in common with many others (e.g., Angel and Clarke, 1975; Kenshalo et al., 1980; Apkarian and Shi, 1994; Montagne-Clavel and Olivéras, 1995), we encountered many more thalamic cells that were LT responsive than HT responsive. We found that LT responses of thalamic neurons in naïve animals appeared more susceptible to ULF inhibition than HT responses—91% of LT responses compared to only 44% of HT responses were significantly attenuated by ULF current. As highlighted above, interpretation of this result mandates some caution due to the small number of HT cells compared to the LT group.

Our earlier findings with ULF current applied to dorsal roots indicated a preference for inhibition of evoked signals carried by LT fibers (Jones et al., 2021), a result that is supported by these observations on thalamic responses with epidural ULF current. We must then consider the significance of this in terms of the types of fibers likely to contribute to the generation and maintenance of chronic pain. To further investigate this, we examined the effect of epidural ULF neuromodulation on thalamic activity in the SNL neuropathic pain model.

Sensory afferent fibers that convey signals from LT receptors are predominantly (though not exclusively—see Liljencrantz and Olausson, 2014) large, myelinated, fast-conducting Aβ-type fibers. Conversely, signals generated by high-intensity stimuli are usually carried by smaller diameter, weakly myelinated Aδ and non-myelinated C type fibers. Our previous work showed that these fibers required higher current amplitudes to inhibit, and our present findings further support this. Traditional dogma holds that many aspects of pathological chronic pain should be mediated by signals originating in the fibers that are responsible for conveying pain-related signals under normal, healthy conditions (i.e., those Aδ- or C-type afferents with typically higher thresholds both for activation and for inhibition by ULF current). The animal findings present a possibly counter-intuitive picture, as human pain patients receiving ULF treatment demonstrated no loss of normal sensation but did experience a significant improvement in chronic pain (Jones et al., 2021).

To better understand this result, it is instructive to consider alternative roles for Aβ fibers in signaling pain. While Aβ-type afferents are less readily associated with nociception, there is plentiful evidence of a population of receptors with fast-conducting Aβ-type fibers that encode HT, nociceptive stimuli (Burgess and Perl, 1967; Lynn and Carpenter, 1982; Djouhri and Lawson, 2004; Fang et al., 2005; Devor, 2009). It seems likely that a proportion of such Aβ nociceptors could contribute to HT responses of some thalamic cells. If this is indeed the case, it may explain why some HT thalamic responses (4 of 9 cells tested in this study) could be diminished by ULF current, possibly affecting conduction in large, myelinated fibers. Additionally, Aβ nociceptors undergo specific changes in action potential dynamics in some pain models (e.g., Wu and Henry, 2009). Such changes appear to manifest only at later time points (up to 2 months after induction of the model) and therefore seem likely to reflect changes that are important in the chronification of disease, as may be seen in human patients.

Another important role for Aβ fibers in the development of chronic pain is central sensitisation, whereby facilitation of responses to primary afferent inputs occurs within the central nervous system (Woolf, 1983). Such facilitation can be produced by an intense input from nociceptors, acutely activated by tissue injury (i.e., in conditions of nociceptive pain; see Woolf, 2011). However, there seems little doubt that central sensitisation may also arise due to a prolonged afferent barrage into the CNS provided by chronic ectopic spontaneous activity of sensory fibers in a neuropathic state (see further discussion below). The relative contributions of abnormal spontaneous activity in A- and C-type fibers to the different aspects of chronic pain remains the subject of debate (see North et al., 2018; Raja et al., 2020). Ectopic firing in A fibers seems likely to underly the phenomenon of touch-evoked allodynia, as selective peripheral block of A-fibers in human patients (Campbell et al., 1988) or interruption of ipsilateral Aβ fiber-specific dorsal column pathways in animals (Sun et al., 2001) alleviates allodynia. Pathological conditions may cause some A fibers to remain ectopically active on a chronic timescale, whereby they then contribute significantly to maintenance of a sensitized state. Additionally, there is a possibility of phenotypic switch of LT Aβ fibers or the neural circuits that they feed into, such that they begin to serve a nociceptive function. Injured A fibers have been shown to begin synthesizing peptides normally associated only with nociceptive sensory afferent fibers, such as substance P (Noguchi et al., 1994; Noguchi et al., 1995; Neumann et al., 1996; Weissner et al., 2006) and calcitonin gene-related peptide (Miki et al., 1998a; Ma et al., 1999). The significance of this lies in a novel ability acquired by Aβ fibers to drive a different type of activity in neurons of the spinal dorsal horn and dorsal column nuclei by releasing pain-related transmitters. This change may explain the nociceptive responses to normally innocuous stimuli that characterize many forms of chronic pain (Pitcher and Henry, 2004), though the phenotypic switch theory has been questioned (Hughes et al., 2007). Another mechanism by which Aβ fiber input can functionally switch from normal mechanosensation to nociceptive-like drive is through disinhibition, which disproportionately affects excitatory neurons (Lee et al., 2019).

Direct investigation of a specific Aβ mechanism was outside of the scope of this study, but we utilized the SNL model to examine the pathological state of thalamic activity levels and the effect of ULF current thereon. Notably, the spontaneous afferent fiber activity seen at early time points after nerve injury occurs almost exclusively in A-fibers (Liu X. et al., 2000; Liu C. N. et al., 2000). SNL is a well-studied model of pain, and the results we present here lend support for an effect of ULF current upon large-diameter fibers. Following experimental axotomy-type nerve lesioning, spontaneous activity in sensory fibers commences soon after the injury. The incidence of firing, and the fiber types which become active, appear to depend to some extent on the model. However, most studies agree that firing in A fibers predominates, with very few spontaneous C fibers, and very low rates of discharge in those C fibers that do show activity (Govrin-Lippmann and Devor, 1978; Wall and Devor, 1983; Kajander and Bennett, 1992; Michaelis et al., 1995; Tal et al., 1999).

Spontaneous activity begins as early as 3 h following axotomy (Kirk, 1974), increases rapidly, reaching a peak within 3 days (Liu X. et al., 2000; Ma et al., 2003) and declines thereafter, but is maintained at a more modest level for weeks. This intense abnormal barrage, continued over several days, is likely to activate at least some elements of the central sensitisation process at multiple sites in the pathway of the signals, including the spinal dorsal horn (Woolf, 1983), gracile nucleus (Miki et al., 1998b; Miki et al., 2000; Wang et al., 2004; Fukuoka et al., 2015) and thalamus itself (Wang et al., 2020). In the sensitized state, interruption of the driving input (i.e., the afferent impulse barrage) may not result in immediate complete cessation or normalization of neuronal firing that has been pathologically elevated for many hours or days, but may nevertheless attenuate it. Our results align with this idea. This provides a possible explanation for the lack of complete inhibition of thalamic cell firing by short-term ULF current in SNL animals.

An alternative interpretation of the observation that ULF current did not silence thalamic neurone firing completely is that the magnitude of the ULF current was insufficient to inhibit conduction of all pathological spontaneous impulses arriving at the spinal cord. To reiterate, the number of spontaneously active fibers observed in the first week after L5 spinal root axotomy is typically very high compared to sham-operated or naïve animals. Liu X. et al. (2000) reported spontaneous fibers in 56% of the filaments they examined; Ma et al. (2003) found an incidence of 89%; our own unpublished findings indicate a similar high incidence of ectopic activity in axotomized fibers. It seems unlikely that ULF current at intensities we used would inhibit conduction in every active fiber throughout the entire L5 spinal root or surrounding neural tissue. The current was applied epidurally in these experiments and there is some separation in the form of dura and cerebrospinal fluid between the electrodes and the underlying neural tissue. To achieve an electric field sufficient to inhibit conduction across the entire root may necessitate increasing the current to a level where even a slowly ramped rise could cause undesired activation of some fibers.

The first week after an injury is a relatively short period of time, and it is established that activity in injured A fibers falls to a lower level as the model matures. However, some degree of spontaneous activity has been shown to persist for many weeks after the initial injury, at which point the model may be regarded as more representative of a chronic neuropathic condition. In our experiments, we showed greatly increased levels of ongoing activity in thalamic cells in the first week after SNL injury, much of which was susceptible to reduction by ULF current applied at lumbar spinal level. Whilst it was not possible to determine fully the physiological role of the thalamic cells we recorded from here (because of deafferentation in the SNL animals), nevertheless ULF current was able to restore thalamic activity to a level similar to that observed in normal uninjured animals (see Tables 2, 3). This finding suggests that such ectopic activity, arising in damaged afferent fibers, can drive ongoing activity in many normally quiescent thalamic neurons, and that ULF current applied at the spinal level is capable of reducing the level of that activity. We cannot be certain about the exact site of action of ULF neuromodulation. We targeted application toward the side of the spinal cord ipsilateral to the nerve injury, at the level where the injured root joins. The reduction of neuronal firing in VPL nucleus may well represent a composite result of inhibitory effects on an afferent barrage in both primary sensory afferents approaching the root entry zone and on fibers ascending in the dorsal columns.

The work here is consistent with our previous experiments, whereby ULF inhibits a wide range of neural signals (Jones et al., 2021). In this work, we continue to expand on the abilities of ULF to inhibit neural signals associated with pain. The development, mechanisms, and maintenance of chronic pain, involving many peripheral and central mechanisms, are still widely debated. Regardless of those particular mechanisms and pathways, in this work, we showed that ULF applied to the epidural space of the spinal cord can have a marked effect on supraspinal signals both in non-pathologic (evoked footshock) and pathologic (SNL) models. Thus, epidural ULF application in a pathological pain model demonstrated a normalizing effect on thalamic activity to resemble more closely the latent activity pattern of the thalamus.

## Data Availability

The data generated for the study are largely included in the article and Supplementary Material. Further inquiries can be directed to the corresponding author.

## References

[ref1] Angel A. Clarke K. A. (1975). An analysis of the representation of the forelimb in the ventrobasal thalamic complex of the albino rat. J. Physiol. 249, 399–423. doi: 10.1113/jphysiol.1975.sp011022, PMID: 1177098 PMC1309581

[ref2] Apkarian A. V. Shi T. (1994). Squirrel monkey lateral thalamus. I. Somatic nociresponsive neurons and their relation to spinothalamic terminals. J. Neurosci. 14, 6779–6795. doi: 10.1523/JNEUROSCI.14-11-06779.1994, PMID: 7965079 PMC6577228

[ref3] Burgess P. R. Perl E. R. (1967). Myelinated afferent fibers responding specifically to noxious stimulation of the skin. J. Physiol. 190, 541–562. doi: 10.1113/jphysiol.1967.sp008227, PMID: 6051786 PMC1365427

[ref4] Campbell J. N. Raja S. N. Meyer R. A. Mackinnon S. E. (1988). Myelinated afferents signal the hyperalgesia associated with nerve injury. Pain 32, 89–94. doi: 10.1016/0304-3959(88)90027-9, PMID: 3340426

[ref5] Casey K. L. Morrow T. J. (1983). Ventral posterior thalamic neurons differentially responsive to noxious stimulation of the awake monkey. Science 221, 675–677. doi: 10.1126/science.6867738, PMID: 6867738

[ref6] Chung J. M. Lee K. H. Surmeier D. J. Sorkin L. S. Kim J. Willis W. D. (1986). Response characteristics of neurons in the ventral posterior lateral nucleus of the monkey thalamus. J. Neurophysiol. 56, 370–390. doi: 10.1152/jn.1986.56.2.370, PMID: 3760926

[ref7] Cohen S. P. Vase L. Hooten W. M. (2021). Chronic pain: an update on burden, best practices, and new advances. Lancet 397, 2082–2097. doi: 10.1016/S0140-6736(21)00393-7, PMID: 34062143

[ref8] Dahlhamer J. Lucas J. Zelaya C. Nahin R. Mackey S. DeBar L.. (2018). Prevalence of chronic pain and high-impact chronic pain among adults - United States, 2016. MMWR Morb. Mortal Wkly. Rep. 67, 1001–1006. doi: 10.15585/mmwr.mm6736a2, PMID: 30212442 PMC6146950

[ref9] Deer T. Slavin K. V. Amirdelfan K. North R. B. Burton A. W. Yearwood T. L.. (2018). Success using neuromodulation with BURST (SUNBURST) study: results from a prospective, randomized controlled trial using a novel burst waveform. Neuromodulation 21, 56–66. doi: 10.1111/ner.12698, PMID: 28961366

[ref10] Devor M. (2009). Ectopic discharge in Abeta afferents as a source of neuropathic pain. Exp. Brain Res. 196, 115–128. doi: 10.1007/s00221-009-1724-6, PMID: 19242687

[ref11] Djouhri L. Lawson S. N. (2004). Ab-fiber nociceptive primary afferent neurons: a review of incidence and properties in relation to other afferent A-fiber neurons in mammals. Brain Res. Brain Res. Rev. 46, 131–145. doi: 10.1016/j.brainresrev.2004.07.015, PMID: 15464202

[ref12] Fang X. McMullan S. Lawson S. N. Djouhri L. (2005). Electrophysiological differences between nociceptive and non-nociceptive dorsal root ganglion neurones in the rat in vivo. J. Physiol. 565, 927–943. doi: 10.1113/jphysiol.2005.086199, PMID: 15831536 PMC1464557

[ref13] Fayaz A. Croft P. Langford R. M. Donaldson L. J. Jones G. T. (2016). Prevalence of chronic pain in the UK: a systematic review and meta-analysis of population studies. BMJ Open 6:e010364. doi: 10.1136/bmjopen-2015-010364, PMID: 27324708 PMC4932255

[ref14] Finnerup N. B. Kuner R. Jensen T. S. (2021). Neuropathic pain: from mechanisms to treatment. Physiol. Rev. 101, 259–301. doi: 10.1152/physrev.00045.2019, PMID: 32584191

[ref15] Fischer T. Z. Tan A. M. Waxman S. G. (2009). Thalamic neuron hyperexcitability and enlarged receptive fields in the STZ model of diabetic pain. Brain Res. 1268, 154–161. doi: 10.1016/j.brainres.2009.02.063, PMID: 19285053

[ref16] Fukuoka T. Miyoshi K. Noguchi K. (2015). De novo expression of Nav1.7 in injured putative proprioceptive afferents: multiple tetrodotoxin-sensitive sodium channels are retained in the rat dorsal root after spinal nerve ligation. Neuroscience 284, 693–706. doi: 10.1016/j.neuroscience.2014.10.027, PMID: 25453779

[ref17] Gautron M. Guilbaud G. (1982). Somatic responses of ventrobasal thalamic neurons in polyarthritic rats. Brain Res. 237, 459–471. doi: 10.1016/0006-8993(82)90457-7, PMID: 7083006

[ref18] GBD 2019 Diseases and Injuries Collaborators (2020). Global burden of 369 diseases and injuries in 204 countries and territories, 1990-2019: a systematic analysis for the Global Burden of Disease Study 2019. Lancet 396, 1204–1222. doi: 10.1016/S0140-6736(20)30925-9. Erratum in: Lancet 2020;396(10262):1562. doi: 10.1016/S0140-6736(20)32226-1, PMID: 33069326 PMC7567026

[ref19] Govrin-Lippmann R. Devor M. (1978). Ongoing activity in severed nerves: source and variation with time. Brain Res. 159, 406–410. doi: 10.1016/0006-8993(78)90548-6, PMID: 215270

[ref20] Guilbaud G. Benoist J. M. Jazat F. Gautron M. (1990). Neuronal responsiveness in the ventrobasal thalamic complex of rats with an experimental peripheral mononeuropathy. J. Neurophysiol. 64, 1537–1554. doi: 10.1152/jn.1990.64.5.1537, PMID: 2283540

[ref21] Guilbaud G. Kayser V. Benoist J. M. Gautron M. (1986). Modifications in the responsiveness of rat ventrobasal thalamic neurons at different stages of carrageenin-produced inflammation. Brain Res. 385, 86–98. doi: 10.1016/0006-8993(86)91550-7, PMID: 3094832

[ref22] Guilbaud G. Peschanski M. Gautron M. Binder D. (1980). Neurons responding to noxious stimulation in VB complex and caudal adjacent regions in the thalamus of the rat. Pain 8, 303–318. doi: 10.1016/0304-3959(80)90076-7, PMID: 7402691

[ref23] Hains B. C. Saab C. Y. Waxman S. G. (2005). Changes in electrophysiological properties and sodium channel Nav1.3 expression in thalamic neurons after spinal cord injury. Brain 128, 2359–2371. doi: 10.1093/brain/awh623, PMID: 16109750

[ref24] Hains B. C. Saab C. Y. Waxman S. G. (2006). Alterations in burst firing of thalamic VPL neurons and reversal by Na(v)1.3 antisense after spinal cord injury. J. Neurophysiol. 95, 3343–3352. doi: 10.1152/jn.01009.2005, PMID: 16481457

[ref25] Harris J. Merrill D. R. Kahn F. Sullivan A. Ackermann D. M. (2022). Response to biphasic ultra-low-frequency (ULF^™^) waveform in sheep sonfirms long-term safety. Oral communication at North American Neuromodulation Society Annual Congress, abstract in press.

[ref26] Hughes D. I. Scott D. T. Riddell J. S. Todd A. J. (2007). Upregulation of substance P in low-threshold myelinated afferents is not required for tactile allodynia in the chronic constriction injury and spinal nerve ligation models. J. Neurosci. 27, 2035–2044. doi: 10.1523/JNEUROSCI.5401-06.2007, PMID: 17314299 PMC1828212

[ref27] Jones M. G. Rogers E. R. Harris J. P. Sullivan A. Ackermann D. M. Russo M.. (2021). Neuromodulation using ultra low frequency current waveform reversibly blocks axonal conduction and chronic pain. Sci. Transl. Med. 13, 1–12. doi: 10.1126/scitranslmed.abg9890, PMID: 34433642

[ref28] Kajander K. C. Bennett G. J. (1992). Onset of a painful peripheral neuropathy in rat: a partial and differential deafferentation and spontaneous discharge in A beta and A delta primary afferent neurons. J. Neurophysiol. 68, 734–744. doi: 10.1152/jn.1992.68.3.734, PMID: 1331353

[ref29] Kapural L. Yu C. Doust M. W. Gliner B. E. Vallejo R. Sitzman B. T.. (2015). Novel 10-kHz high-frequency therapy (HF10 Therapy) is superior to traditional low-frequency spinal cord stimulation for the treatment of chronic back and leg pain: the SENZA-RCT randomized controlled trial. Anesthesiology 123, 851–860. doi: 10.1097/ALN.0000000000000774, PMID: 26218762

[ref30] Kenshalo D. R. Jr. Giesler G. J. Jr. Leonard R. B. Willis W. D. (1980). Responses of neurons in primate ventral posterior lateral nucleus to noxious stimuli. J. Neurophysiol. 43, 1594–1614. doi: 10.1152/jn.1980.43.6.1594, PMID: 7411178

[ref31] Kirk E. J. (1974). Impulses in dorsal spinal nerve rootlets in cats and rabbits arising from dorsal root ganglia isolated from the periphery. J. Comp. Neurol. 155, 165–175. doi: 10.1002/cne.901550203, PMID: 4827008

[ref32] Knotkova H. Hamani C. Sivanesan E. Le Beuffe M. F. E. Moon J. Y. Cohen S. P.. (2021). Neuromodulation for chronic pain. Lancet 397, 2111–2124. doi: 10.1016/S0140-6736(21)00794-7, PMID: 34062145

[ref33] Kuner R. Flor H. (2016). Structural plasticity and reorganisation in chronic pain. Nat. Rev. Neurosci. 18, 20–30. doi: 10.1038/nrn.2016.162, PMID: 27974843

[ref34] Lee K. Y. Ratté S. Prescott S. A. (2019). Excitatory neurons are more disinhibited than inhibitory neurons by chloride dysregulation in the spinal dorsal horn. eLife 8:e49753. doi: 10.7554/eLife.49753, PMID: 31742556 PMC6887484

[ref35] Lempka S. F. Patil P. G. (2018). Innovations in spinal cord stimulation for pain. Curr. Opin. Biomed. Eng. 8, 51–60. doi: 10.1016/j.cobme.2018.10.005, PMID: 30911705 PMC6430588

[ref36] Liljencrantz J. Olausson H. (2014). Tactile C fibers and their contributions to pleasant sensations and to tactile allodynia. Front. Behav. Neurosci. 8:37. doi: 10.3389/fnbeh.2014.00037, PMID: 24639633 PMC3944476

[ref37] Liu X. Eschenfelder S. Blenk K. H. Jänig W. Häbler H. (2000). Spontaneous activity of axotomized afferent neurons after L5 spinal nerve injury in rats. Pain 84, 309–318. doi: 10.1016/s0304-3959(99)00211-0, PMID: 10666536

[ref38] Liu C. N. Wall P. D. Ben-Dor E. Michaelis M. Amir R. Devor M. (2000). Tactile allodynia in the absence of C-fiber activation: altered firing properties of DRG neurons following spinal nerve injury. Pain 85, 503–521. doi: 10.1016/S0304-3959(00)00251-7, PMID: 10781925

[ref39] Lynn B. Carpenter S. E. (1982). Primary afferent units from the hairy skin of the rat hind limb. Brain Res. 238, 29–43. doi: 10.1016/0006-8993(82)90768-5, PMID: 6282398

[ref40] Ma W. Ramer M. S. Bisby M. A. (1999). Increased calcitonin gene-related peptide immunoreactivity in gracile nucleus after partial sciatic nerve injury: age-dependent and originating from spared sensory neurons. Exp. Neurol. 159, 459–473. doi: 10.1006/exnr.1999.7149, PMID: 10506517

[ref41] Ma C. Shu Y. Zheng Z. Chen Y. Yao H. Greenquist K. W.. (2003). Similar electrophysiological changes in axotomized and neighboring intact dorsal root ganglion neurons. J. Neurophysiol. 89, 1588–1602. doi: 10.1152/jn.00855.2002, PMID: 12612024

[ref42] Melzack R. Wall P. D. (1965). Pain mechanisms: a new theory. Science 150, 971–979. doi: 10.1126/science.150.3699.971, PMID: 5320816

[ref43] Michaelis M. Blenk K. H. Jänig W. Vogel C. (1995). Development of spontaneous activity and mechanosensitivity in axotomized afferent nerve fibers during the first hours after nerve transection in rats. J. Neurophysiol. 74, 1020–1027. doi: 10.1152/jn.1995.74.3.1020, PMID: 7500128

[ref44] Miki K. Fukuoka T. Tokunaga A. Noguchi K. (1998a). Calcitonin gene-related peptide increase in the rat spinal dorsal horn and dorsal column nucleus following peripheral nerve injury: up-regulation in a subpopulation of primary afferent sensory neurons. Neuroscience 82, 1243–1252. doi: 10.1016/s0306-4522(97)00258-3, PMID: 9466443

[ref45] Miki K. Iwata K. Tsuboi Y. Morimoto T. Kondo E. Dai Y.. (2000). Dorsal column-thalamic pathway is involved in thalamic hyperexcitability following peripheral nerve injury: a lesion study in rats with experimental mononeuropathy. Pain 85, 263–271. doi: 10.1016/s0304-3959(99)00279-1, PMID: 10692627

[ref46] Miki K. Iwata K. Tsuboi Y. Sumino R. Fukuoka T. Tachibana T.. (1998b). Responses of dorsal column nuclei neurons in rats with experimental mononeuropathy. Pain 76, 407–415. doi: 10.1016/S0304-3959(98)00073-6, PMID: 9718259

[ref47] Montagne-Clavel J. Olivéras J. L. (1995). Does barbiturate anesthesia modify the neuronal properties of the somatosensory thalamus? A single-unit study related to nociception in the awake-pentobarbital-treated rat. Neurosci. Lett. 196, 69–72. doi: 10.1016/0304-3940(95)11847-p, PMID: 7501260

[ref48] Neumann S. Doubell T. P. Leslie T. Woolf C. J. (1996). Inflammatory pain hypersensitivity mediated by phenotypic switch in myelinated primary sensory neurons. Nature 384, 360–364. doi: 10.1038/384360a0, PMID: 8934522

[ref49] Noguchi K. Dubner R. De Leon M. Senba E. Ruda M. A. (1994). Axotomy induces preprotachykinin gene expression in a subpopulation of dorsal root ganglion neurons. J. Neurosci. Res. 37, 596–603. doi: 10.1002/jnr.490370506, PMID: 7518007

[ref50] Noguchi K. Kawai Y. Fukuoka T. Senba E. Miki K. (1995). Substance P induced by peripheral nerve injury in primary afferent sensory neurons and its effect on dorsal column nucleus neurons. J. Neurosci. 15, 7633–7643. doi: 10.1523/JNEUROSCI.15-11-07633.1995, PMID: 7472514 PMC6578074

[ref51] North R. Y. Lazaro T. T. Dougherty P. M. (2018). Ectopic spontaneous afferent activity and neuropathic pain. Neurosurgery 65, 49–54. doi: 10.1093/neuros/nyy119, PMID: 31076785

[ref52] Patel R. Dickenson A. H. (2016). Neuronal hyperexcitability in the ventral posterior thalamus of neuropathic rats: modality selective effects of pregabalin. J. Neurophysiol. 116, 159–170. doi: 10.1152/jn.00237.2016, PMID: 27098028 PMC4961752

[ref53] Paxinos G. Watson C. (2004). The rat brain in stereotaxic coordinates. 5th Edn. London: Academic Press.

[ref54] Pitcher G. M. Henry J. L. (2004). Nociceptive response to innocuous mechanical stimulation is mediated via myelinated afferents and NK-1 receptor activation in a rat model of neuropathic pain. Exp. Neurol. 186, 173–197. doi: 10.1016/j.expneurol.2003.10.019, PMID: 15026255

[ref55] Raja S. N. Ringkamp M. Guan Y. Campbell J. N. (2020). John J. Bonica award lecture: peripheral neuronal hyperexcitability: the "low-hanging" target for safe therapeutic strategies in neuropathic pain. Pain 161 Suppl 1, S14–S26. doi: 10.1097/j.pain.0000000000001838, PMID: 33090736 PMC7586453

[ref56] Rice A. S. C. Cimino-Brown D. Eisenach J. C. Kontinen V. K. Lacroix-Fralish M. L. Machin I.. (2008). Animal models and the prediction of efficacy in clinical trials of analgesic drugs: a critical appraisal and call for uniform reporting standards. Pain 139, 243–247. doi: 10.1016/j.pain.2008.08.017, PMID: 18814968

[ref57] Roza C. Bernal L. (2022). Electrophysiological characterization of ectopic spontaneous discharge in axotomized and intact fibers upon nerve transection: a role in spontaneous pain? Pflugers Arch. 474, 387–396. doi: 10.1007/s00424-021-02655-7, PMID: 35088129

[ref58] Scholl L. Seth P. Kariisa M. Wilson N. Baldwin G. (2018). Drug and Opioid-Involved Overdose Deaths - United States, 2013-2017. MMWR Morb. Mortal Wkly. Rep. 67, 1419–1427. doi: 10.15585/mmwr.mm675152e1, PMID: 30605448 PMC6334822

[ref59] Shealy C. N. Mortimer J. T. Reswick J. B. (1967). Electrical inhibition of pain by stimulation of the dorsal columns: preliminary clinical report. Anesth. Analg. 46, 489–491. doi: 10.1213/00000539-196707000-00025, PMID: 4952225

[ref60] Sun H. Ren K. Zhong C. M. Ossipov M. H. Malan T. P. Lai J.. (2001). Nerve injury-induced tactile allodynia is mediated via ascending spinal dorsal column projections. Pain 90, 105–111. doi: 10.1016/s0304-3959(00)00392-4, PMID: 11166976

[ref61] Syré P. P. Weisshaar C. L. Winkelstein B. A. (2014). Sustained neuronal hyperexcitability is evident in the thalamus after a transient cervical radicular injury. Spine 39, E870–E877. doi: 10.1097/BRS.0000000000000392, PMID: 24827526

[ref62] Tal M. Wall P. D. Devor M. (1999). Myelinated afferent fiber types that become spontaneously active and mechanosensitive following nerve transection in the rat. Brain Res. 824, 218–223. doi: 10.1016/s0006-8993(99)01190-7, PMID: 10196451

[ref63] Taylor R. S. Desai M. J. Rigoard P. Taylor R. J. (2014). Predictors of pain relief following spinal cord stimulation in chronic back and leg pain and failed back surgery syndrome: a systematic review and meta-regression analysis. Pain Pract. 14, 489–505. doi: 10.1111/papr.12095, PMID: 23834386 PMC4238825

[ref64] Verma N. Rietman M. Merrill D. Sullivan A. Harris J. (2025). O107 Long-term safety of ultra-low-frequency waveform shown in large animals. Neuromodulation 28:S131. doi: 10.1016/j.neurom.2024.09.208

[ref65] Vos B. P. Benoist J. M. Gautron M. Guilbaud G. (2000). Changes in neuronal activities in the two ventral posterior medial thalamic nuclei in an experimental model of trigeminal pain in the rat by constriction of one infraorbital nerve. Somatosens. Mot. Res. 17, 109–122. doi: 10.1080/08990220050020535, PMID: 10895882

[ref66] Wall P. D. Devor M. (1983). Sensory afferent impulses originate from dorsal root ganglia as well as from the periphery in normal and nerve injured rats. Pain 17, 321–339. doi: 10.1016/0304-3959(83)90164-1, PMID: 6664680

[ref67] Wang H. Dai Y. Fukuoka T. Yamanaka H. Obata K. Tokunaga A.. (2004). Enhancement of stimulation-induced ERK activation in the spinal dorsal horn and gracile nucleus neurons in rats with peripheral nerve injury. Eur. J. Neurosci. 19, 884–890. doi: 10.1111/j.0953-816x.2004.03203.x, PMID: 15009135

[ref68] Wang Z. Huang S. Yu X. Li L. Yang M. Liang S.. (2020). Altered thalamic neurotransmitters metabolism and functional connectivity during the development of chronic constriction injury induced neuropathic pain. Biol. Res. 53:36. doi: 10.1186/s40659-020-00303-5, PMID: 32843088 PMC7448455

[ref69] Weissner W. Winterson B. J. Stuart-Tilley A. Devor M. Bove G. M. (2006). Time course of substance P expression in dorsal root ganglia following complete spinal nerve transection. J. Comp. Neurol. 497, 78–87. doi: 10.1002/cne.20981, PMID: 16680762 PMC2571959

[ref70] Woolf C. J. (1983). Evidence for a central component of post-injury pain hypersensitivity. Nature 306, 686–688. doi: 10.1038/306686a0, PMID: 6656869

[ref71] Woolf C. J. (2011). Central sensitization: implications for the diagnosis and treatment of pain. Pain 152, S2–S15. doi: 10.1016/j.pain.2010.09.030, PMID: 20961685 PMC3268359

[ref72] Wu Q. Henry J. L. (2009). Delayed onset of changes in soma action potential genesis in nociceptive A-beta DRG neurons in vivo in a rat model of osteoarthritis. Mol. Pain 5:57. doi: 10.1186/1744-8069-5-57, PMID: 19785765 PMC2761878

[ref73] Yang F. Anderson M. He S. Stephens K. Zheng Y. Chen Z.. (2018). Differential expression of voltage-gated sodium channels in afferent neurons renders selective neural block by ionic direct current. Sci. Adv. 4:eaaq1438. doi: 10.1126/sciadv.aaq1438, PMID: 29651458 PMC5895440

