# Supplementary figures and images for: Spinal neuromodulation using ultra low frequency waveform inhibits sensory signaling to the thalamus and preferentially reduces aberrant firing of thalamic neurons in a model of neuropathic pain
Source: Front Neurosci. 2025 Jan 17;18:1512950. doi: 10.3389/fnins.2024.1512950 (PMC11783389; doi:10.3389/fnins.2024.1512950)

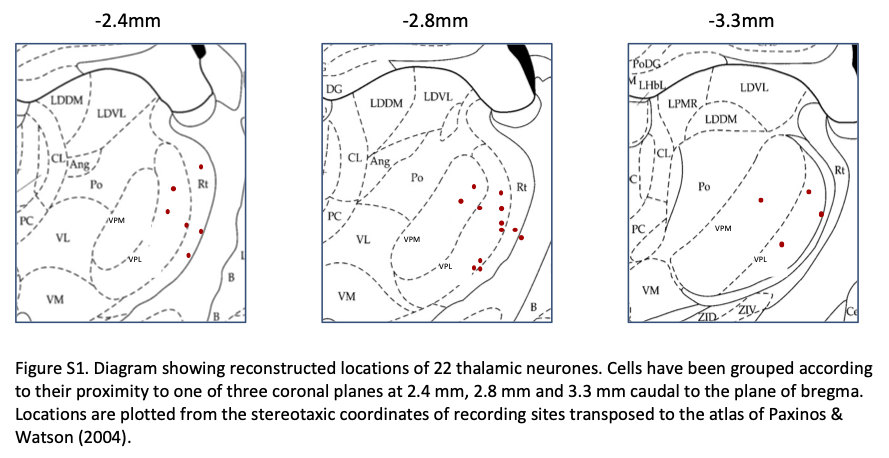

Supplement: Supplementary file 1 [file Image_1.tiff]

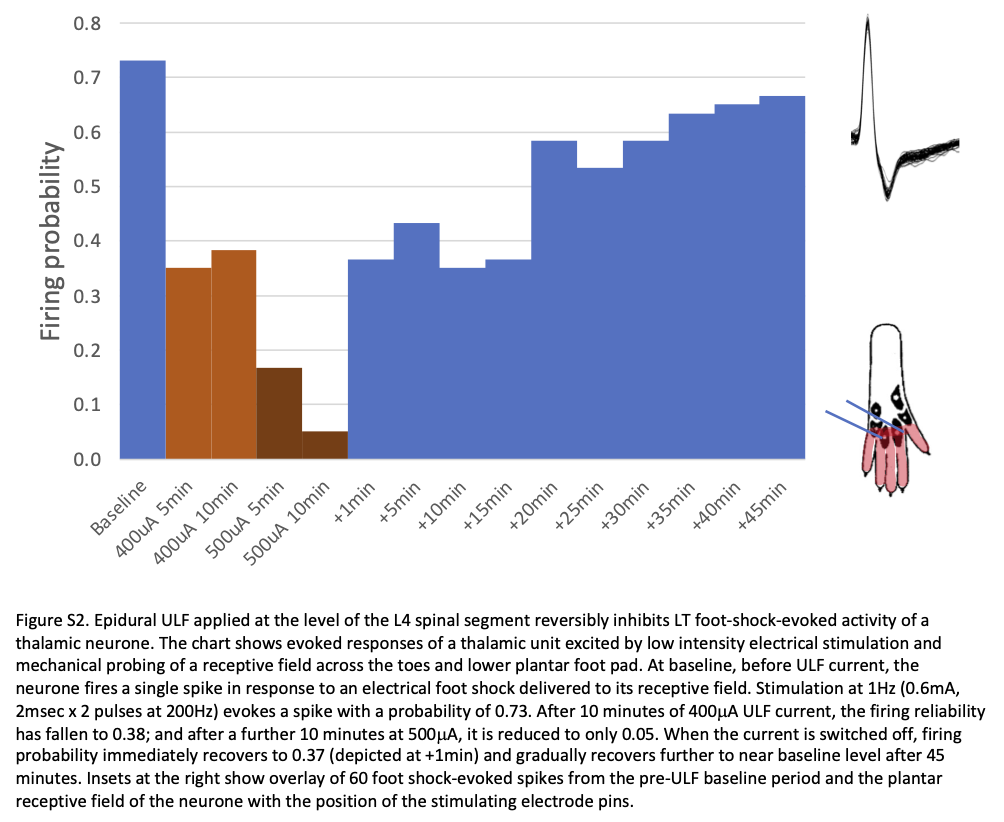

Supplement: Supplementary file 2 [file Image_2.tiff]

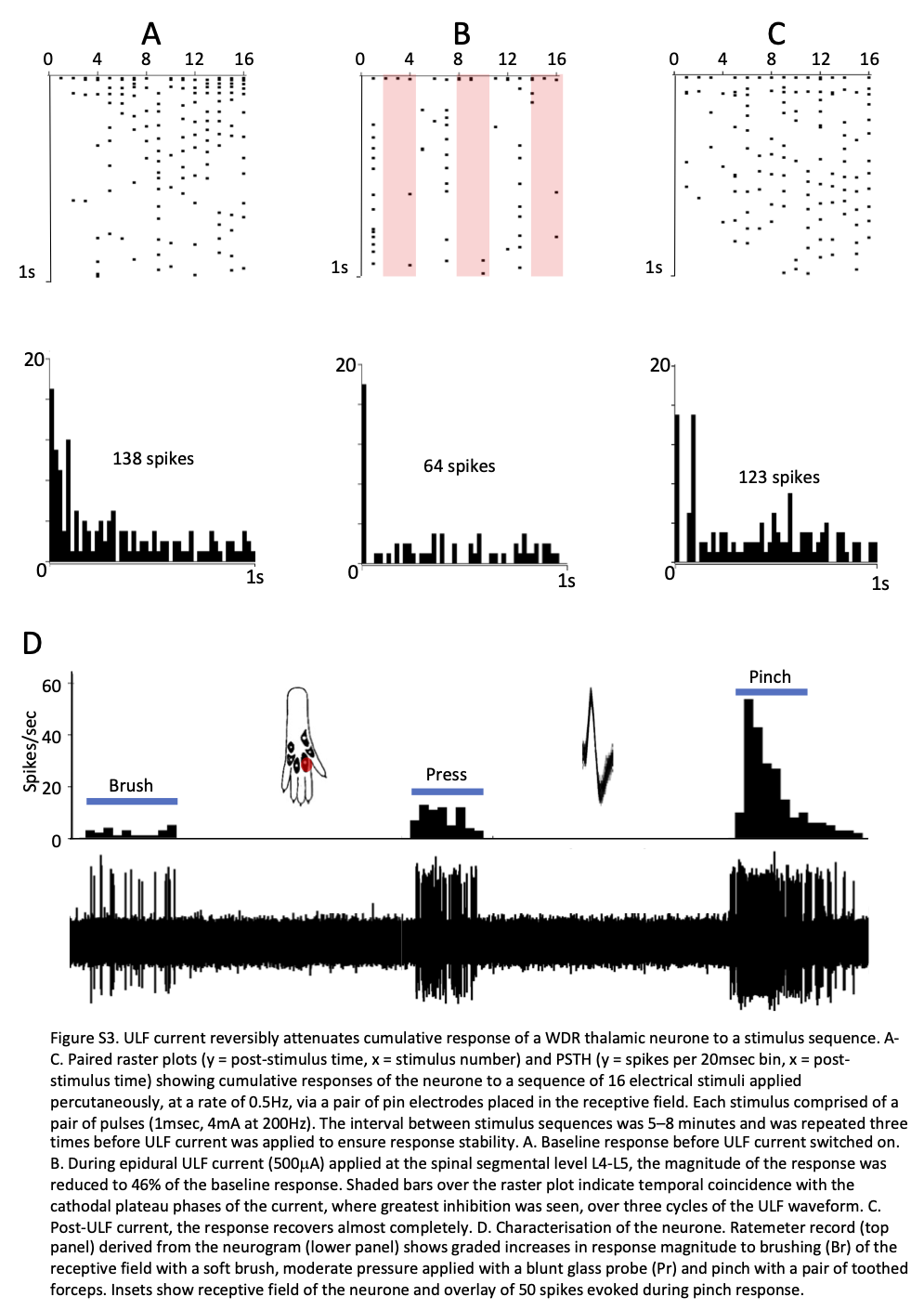

Supplement: Supplementary file 3 [file Image_3.tiff]

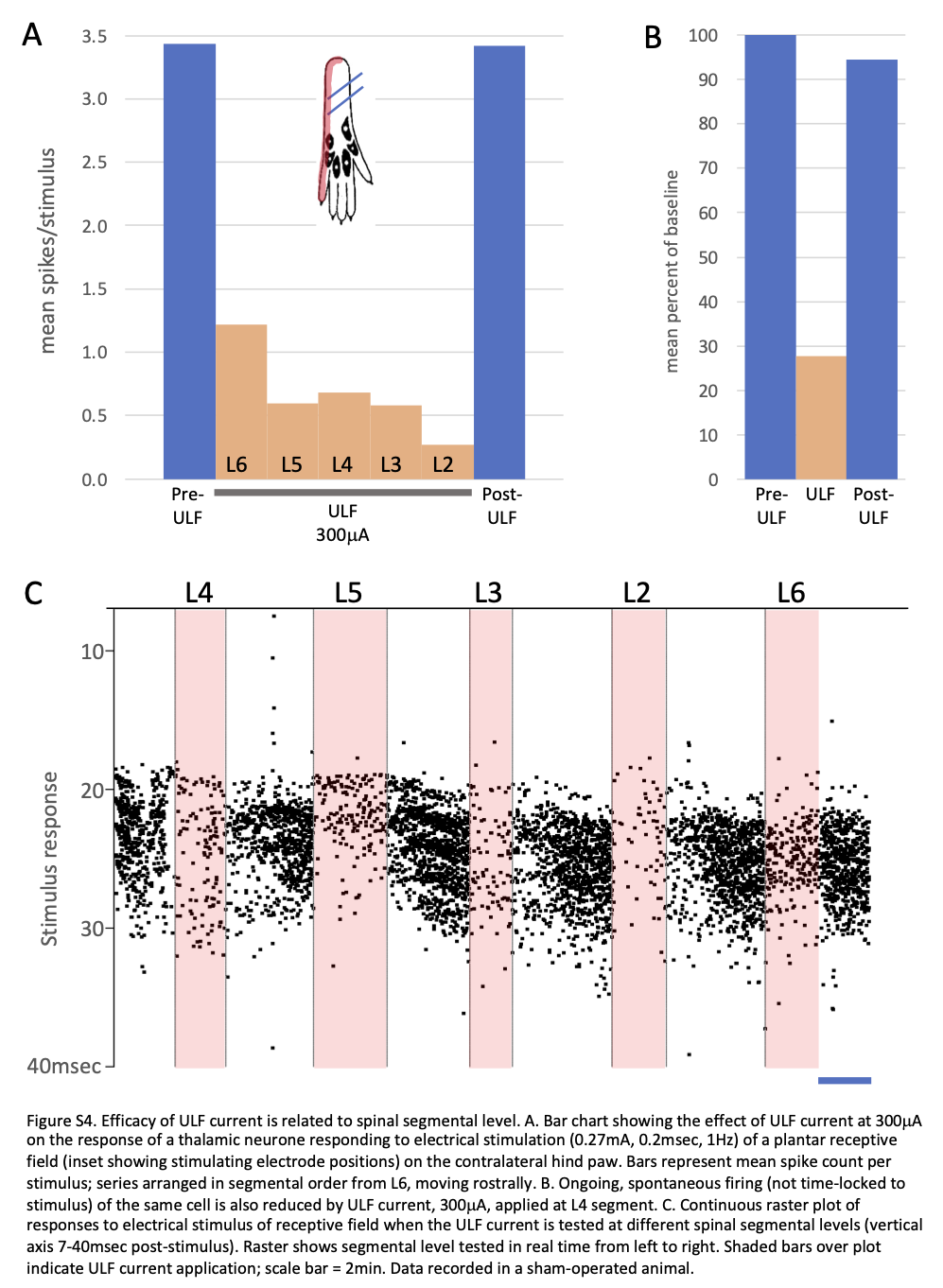

Supplement: Supplementary file 4 [file Image_4.tiff]

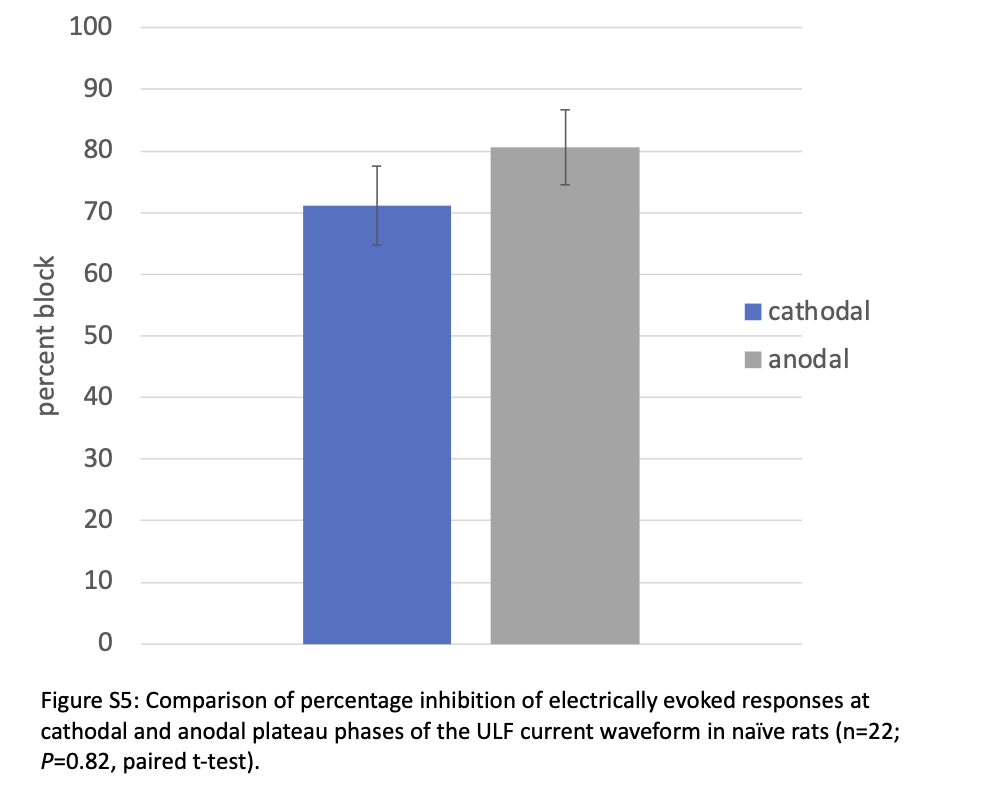

Supplement: Supplementary file 5 [file Image_5.tiff]

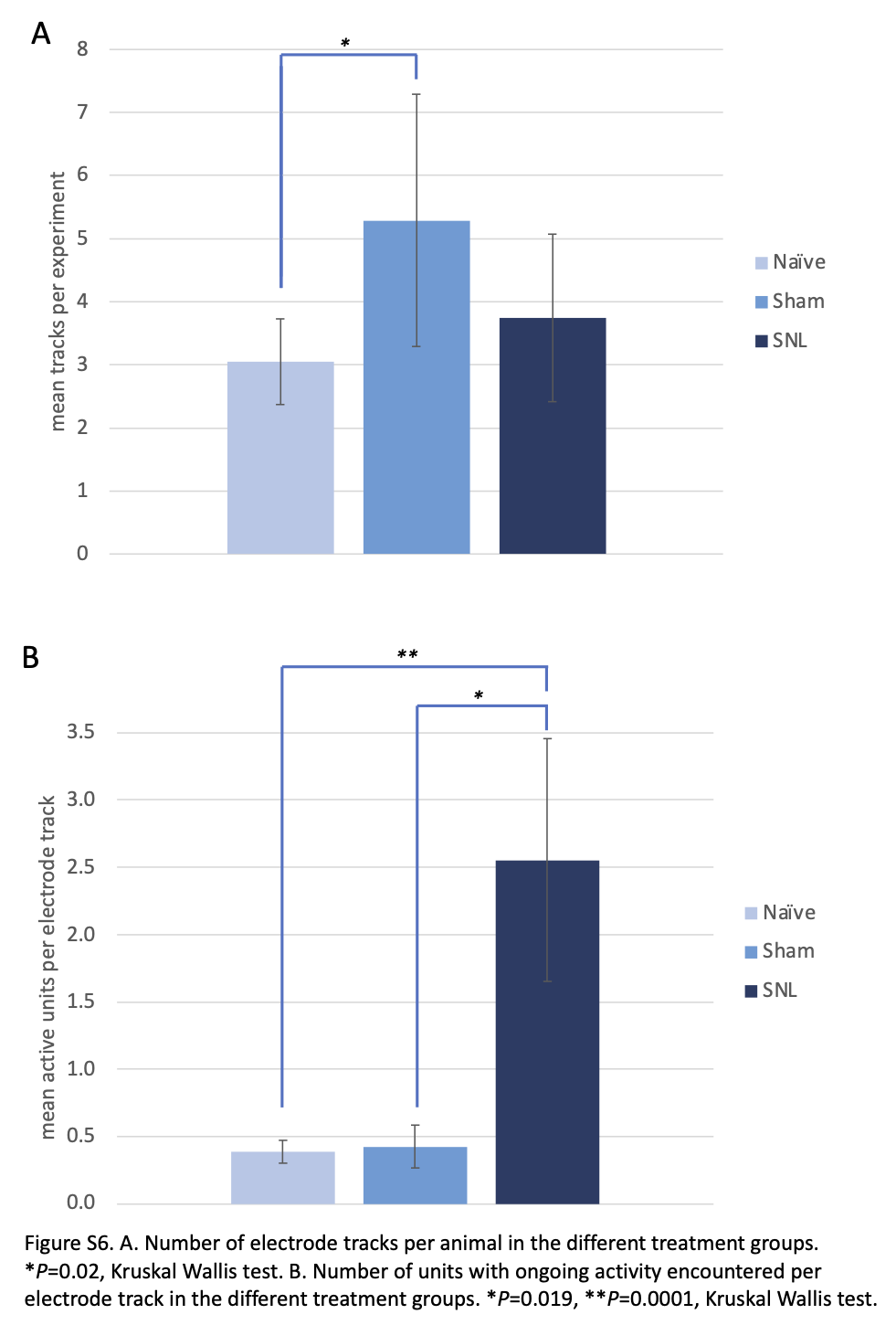

Supplement: Supplementary file 6 [file Image_6.tiff]
